# Photoactivatable Platinum-Based Anticancer Drugs: Mode of Photoactivation and Mechanism of Action

**DOI:** 10.3390/molecules25215167

**Published:** 2020-11-06

**Authors:** Ziwen Dai, Zhigang Wang

**Affiliations:** 1 School of Pharmaceutical Sciences, Health Science Center, Shenzhen University, Shenzhen 518055, China; ziwen.dai@szu.edu.cn; 2 College of Materials Science and Engineering, Shenzhen University, Shenzhen 518055, China; 3 Key Laboratory of Optoelectronic Devices and Systems of Ministry of Education and Guangdong Province, College of Optoelectronic Engineering, Shenzhen University, Shenzhen 518060, China

**Keywords:** anticancer, photoactivatable platinum drugs, photoactivate chemotherapy, prodrugs

## Abstract

Platinum-based anticancer drugs are a class of widely used agents in clinical cancer treatment. However, their efficacy was greatly limited by their severe side effects and the arising drug resistance. The selective activation of inert platinum-based drugs in the tumor site by light irradiation is able to reduce side effects, and the novel mechanism of action of photoactivatable platinum drugs might also conquer the resistance. In this review, the recent advances in the design of photoactivatable platinum-based drugs were summarized. The complexes are classified according to their mode of action, including photoreduction, photo-uncaging, and photodissociation. The rationale of drug design, dark stability, photoactivation process, cytotoxicity, and mechanism of action of typical photoactivatable platinum drugs were reviewed. Finally, the challenges and opportunities for designing more potent photoactivatable platinum drugs were discussed.

## 1. Introduction

**Figure 1:**
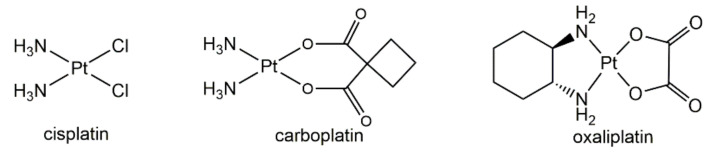
Chemical structure of cisplatin, carboplatin, and oxaliplatin.

In 1965, Rosenberg found that some substances in platinum mesh electrodes could inhibit the cell division in *Escherichia coli*, and the researcher confirmed it was *cis*-diaminedichloroplatinum(II), that is, cisplatin (Figure 1) [1]. Soon, researchers elucidated the mechanism of action of cisplatin killing cells. They found that after entering cells, the chloride on cisplatin would be replaced by water molecules and form hydrated cisplatin [2,3]. Then, the hydrated cisplatin would bind to the N7 position of purines in DNA and formed intra- or inter-strand crosslinks. This inhibited the replication and transcription of DNA, and thus inhibited the cell division and finally initiated the apoptotic pathway to kill cells. After the elucidation of the mechanism, many more researchers swarmed into this field and developed various metal complexes-based anticancer drugs, including platinum, palladium, and other noble metals [4]. In 1978, cisplatin was approved by the U.S. Food and Drug Administration (FDA) for cancer treatment, and since then, cisplatin was applied widely in the treatment of various types of cancers, including lung, ovarian, carcinoma, breast, brain, and neck cancers [5]. However, it was soon found that cisplatin would bring about severe side effects. Meanwhile, some cancers acquired resistance to cisplatin [6,7]. Thus, novel platinum-based anticancer drugs such as carboplatin and oxaliplatin (Figure 1) were developed. Among those new platinum-based anticancer drugs, carboplatin demonstrated definite advantages over cisplatin in decreased side effects, and oxaliplatin was particularly approved for use in colorectal cancer treatment. However, the structures and mechanisms of action of these Pt(II) drugs are similar to that of cisplatin; the problems in cisplatin were also found in these Pt(II) drugs, more or less. Thus, the development of new types of platinum drugs to overcome side effects and resistance issues is highly appealing.

The side effects of platinum drugs arose from the non-specific binding of Pt(II) to biomolecules. They could be minimized if platinum drugs could be selectively activated in the tumor site. To achieve this, one way is to target platinum drugs to the tumor sites to enhance the accumulation of Pt drugs in tumors and decrease the distribution of Pt drugs in normal tissues [8,9,10,11]. Another way is to design inert prodrugs of Pt, which are non-toxic to cells. The prodrugs could be selectively activated to active drugs in the tumors via various endogenous and exogenous stimuli, such as overexpressed enzymes within the tumors, higher H_2_O_2_ concentration, lower pH value, hypoxia, ultrasound, and light [12]. Among the various kinds of activatable platinum-based anticancer complexes, photoactivatable platinum drugs have unique advantages due to the spatial and temporal precision of light [13]. Light is easy to be controlled compared with other endogenous stimuli, such as pH and enzymes. Photodynamic therapy (PDT), which depends on a photosensitizer to generate reactive oxygen species to kill cancer cells under light irradiation, has been used in clinics for the treatment of skin, bladder, and brain cancers [14,15]. The lifetime of ROS is very short, which only leads to cellular damage in the irradiated area, thus minimizing the side effects. Nevertheless, PDT needs oxygen, while most of the solid tumor environment is hypoxic, which limited the therapeutic effects of PDT [16]. The photoactivation of platinum drugs is oxygen-independent, which might be more suitable for hypoxic tumors.

With the rapid development of platinum-based anticancer complexes, several types of photoactivatable Pt(II) and Pt(IV) complexes were prepared, their modes of photoactivation were elucidated, and their antitumor effects were assessed [12]. So far, several excellent reviews have been published, which reported the development and achievement of photoactivatable platinum complexes [17,18,19]. However, no one has done a comprehensive summary on platinum drugs based on their photoactivation mechanism. By understanding the mechanism of photoactivatable platinum complexes, it should be possible to design new photoactivatable platinum complexes in a more rational way. Herein, the photoactivatable platinum drugs based on the classification of the mechanism of photoactivation will be summarized. The complexes will be divided into groups based on their mechanisms of action, including photoreduction, photo-uncaging, and photodissociation. In addition to these types of photoactivatable platinum drugs, dual-action platinum drugs built by a conjugation of PDT agents with platinum drugs also showed enhanced cytotoxicity under irradiation compared with that in the darkness [20,21,22,23,24,25]. This kind of complex will not be discussed in this review, as this review will focus on the platinum complexes that could generate an active form of Pt(II) species upon irradiation. In addition, the delivery of photoactivatable platinum drugs by using nanomaterials such as upconversion nanoparticles (UCNPs), quantum dots (QDs), and lipids is beyond the present scope of this article as well [26,27,28,29,30,31,32]. Readers could get this information in other recent review papers [12,17,33].

## 2. Photoreduction Platinum Complexes

Pt(IV) complexes are natural prodrugs of Pt(II) anticancer drugs. Pt(IV) complexes were prepared by the oxidation of square planar Pt(II) complexes, and two ligands were added to the axial position of the Pt during the oxidation process [34]. The Pt center in Pt(IV) complexes adopted d^6^ electronic configuration, which was kinetically inert toward ligand exchange, minimizing unwanted interactions with nucleophiles [35,36]. After entering cells, Pt(IV) complexes were reduced to its active Pt(II) form by cellular reductants to exert anticancer effects. Thus, the key to developing photoreduction platinum complexes was to shift this spontaneous cellular reduction process to photo-induced reduction. An ideal photoreduction Pt(IV) complex should be kinetically resistant to cellular reduction in the darkness, while upon irradiation, the complex could be rapidly reduced to its active Pt(II) species to kill cancer cells.

### 2.1. The Diiodo Pt(IV) Photoactive Complexes

**Figure 2:**
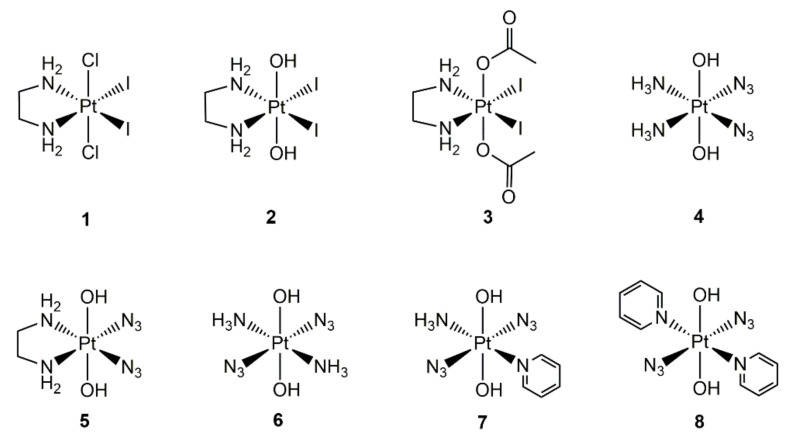
Chemical structures of iodoplatinum(IV)–amines **1**–**3** and diazido Pt(IV) complexes **4**–**8.**

The pioneering work in a photoactivatable Pt(IV) complex was reported by Bednarski and co-workers in 1996 [37]. They incorporated iodine atoms into the coordination sphere of Pt(IV). Due to the small optical electronegativity of iodide, iodoplatinum(IV)–amines complexes showed the ligand-to-metal charge-transfer (LMCT) band at about 400 nm. The photoreaction of Pt(IV) complexes usually originated from LMCT excitation; thus, iodoplatinum(IV)–amines (**1**) complexes (Figure 2) were anticipated to undergo photochemical reductive eliminations by LMCT excitation using visible light around 400 nm. An irradiation of complex **1** at 410 nm led to the formation of new species that can bind to DNA and suppress the growth of tumor cells. The new species were not fully characterized, but they were suggested to be I_2_ and [PtCl_2_(en)] from the results of UV-visible spectroscopy. However, complex **1** was relatively unstable in the presence of serum in the darkness, which limited its further application. To enhance the stability, complexes **2** and **3** (Figure 2) were prepared by replacing the axial chloride in complex **1** with hydroxyl or acetoxyl groups [38]. Both complexes **2** and **3** showed better stability in cell culture medium compared with that of complex **1**. Under irradiation with visible light (>370 nm), complex **2** was photolyzed, but the resultant species were Pt(IV) species instead of active Pt(II) species. Complex **3** can be photolyzed by visible light (>370 nm) to active species that platinated DNA. The IC_50_ values of complex **3** against TCCSUP human bladder cancer cells with or without irradiation were 11.6 ± 1.7 μM and 16.6 ± 1.7 μM, respectively. The phototoxic index (PI, IC_50_ value in the darkness/IC_50_ value with irradiation) was relatively low. The higher toxicity of complex **3** in the darkness could be attributed to the rapid reduction by glutathione (GSH) or ascorbic acid. These iodo–Pt(IV) complexes opened the era of photoactivatable Pt(IV) anticancer drugs. However, the photoactivation process needed a long irradiation time (1.5 h), and the stability of the complexes in the presence of biological reductants in the darkness was poor. These drawbacks limited their application; thus, the further development of this kind of complex as a photoactivatable anticancer agent was discontinued.

### 2.2. The Diazido Pt(IV) Photoactive Complexes

Azido Pt(IV) complexes have been shown to undergo reductive elimination of the azide ligands under irradiation by Vogler′s group in the 1980s [39,40]. Based on the photochemistry of azido Pt(IV) complexes, Sadler and co-workers designed complexes **4** and **5** (Figure 2), in which the iodide was replaced with azide [41]. NMR studies evidenced that complexes **4** and **5** reacted only slowly with GSH over a period of several weeks. The complexes were stable in human blood plasma, and no reactions with 5′-GMP or d(GpG) were observed over a period of one week in the darkness, indicating the excellent dark stability of the complexes against biological molecules. Then, the reactions of complexes **4** and **5** with 5′-GMP or d(GpG) under irradiation were elucidated by NMR spectroscopy. Complex **5** reacted with 5′-GMP (two molar equivalents) in water under irradiation with lower-power blue light (457.9 nm, 15 mW, 20 h) to give photoreduced products [Pt^II^(en)(GMP-N7)_2_]^2+^ and other Pt-GMP species. The reaction of complex **5** with one molar equivalent of d(GpG) in the presence of blue light (457.9 nm, 15 mW, 3.1h) resulted in the formation of [Pt^II^(en){d(GpG)-N7^1^,N7^2^}]^2+^ as a major product. These results indicated that complex **5** was reduced to its active Pt(II) form and bound to the N7 position of guanine. Similar to that of complex **5**, *cis*-[Pt(NH_3_)_2_{d(GpG)-N7^1^,N7^2^}]^2+^ was observed as a product in the reaction of complex **4** with d(GpG) under irradiation. Further investigations confirmed that photoreduction, photosubstitution, and photoisomerization were involved in the photoreactions. Given the excellent dark stability and photoreaction efficiency with guanine of **4** and **5**, their in vitro photocytotoxicity against human bladder cancer cell line 5637 was tested. Both complexes **4** and **5** showed low cytotoxicity in the darkness with IC_50_ values larger than 350 μM. Upon irradiation with a Lamag 40 W UV-lamp (λ = 366 nm) for 6 h, the IC_50_ values of complexes **4** and **5** decreased to 49 and 63 μM, respectively [42]. Moreover, the prodrugs **4** and **5** were equally cytotoxic to 5637 cells and cisplatin-resistant 5637 cells, suggesting that this kind of complex might hold great potential to overcome cisplatin resistance. Further observation of the cellular morphological changes of 5637 cells upon treatment with prodrug **4** with irradiation indicated that the cells underwent an anomalous autophagic mode of cell death in contrast to the apoptotic pathway induced by cisplatin.

Transplatin, the *trans* isomer of cisplatin, is relatively non-toxic, possibly owing to that the geometrical arrangement would prevent the formation of lethal intra-stand GG crosslinks on DNA. However, some *trans*-diamine Pt(IV) complexes were observed to show comparable cytotoxicity to cisplatin [43]. Based on this, Sadler et al. developed complex **6** (Figure 2), the *trans* isomer of complex **4**, as a photoactivatable prodrug [44]. Complex **6** had a higher aqueous solubility and a more intense LMCT band with a longer wavelength than the *cis*-isomer **4**. After irradiation of the water solution of **6** with UVA light (λ = 365 nm, 12 mW) for 1 h, Pt(II) species were detected by NMR spectroscopy. However, even after irradiation for 2 h, the majority of Pt was still in the Pt(IV) state, and it had not been reduced. However, in the presence of two equivalent of 5′-GMP, the photoreduction was much faster, the photoproduced species-formed bis-GMP adduct, *trans*-[Pt(NH_3_)_2_(5′-GMP-N7)_2_]^2+^, was detected only one minute after irradiation, and >75% of complex **6** was converted to the bis-GMP product after 1 h of irradiation. In contrast, irradiation of **4** in the presence of 5′-GMP gave both *cis* and *trans* bis-GMP adducts. Complex **6** was non-toxic (IC_50_ > 288 μM) toward HaCaT cells in the darkness. The cytotoxicity of **6** against HaCaT cells significantly increased (IC_50_ = 156 μM) in the presence of UVA light (5 J/cm^2^, 50 min), which was comparable to that of cisplatin (IC_50_ = 144 μM). Further mechanism studies revealed that the phototoxicity of **6** was correlated with the formation of crosslinks in DNA, which was similar to that of cisplatin. The mechanism of action was different from that of transplatin, indicating that complex **6** was not simply a prodrug of transplatin. These results suggest that the *trans*-diazido Pt(IV) complex was a potent photoactivatable prodrug with high stability in the darkness, fast activation under irradiation, and superior photocytotoxicity.

Encouraged by the outstanding properties of *trans*-diazido Pt(IV) complexes, researchers prepared a series of *trans*-diazido Pt(IV) complexes by varying the amines to different *N*-donor ligands, including aliphatic amines (methylamine, ethylamine, and cyclohexylamine) and aromatic *N*-donors (pyridine, picoline, quinolone, and thiazole) to reveal the structure–activity relationship (SAR) and to screen for more potent photoactivatable Pt(IV) prodrugs [18]. Among them, complex **7** (Figure 2), which had one *trans* pyridine instead of one amine, exhibited superior photocytotoxicity [45]. Complex **7** was remarkably stable in water and did not react with two equivalents of GMP in the darkness over a period of 5 months. In the presence of glutathione (2 equiv.), only 5% of complex **7** was reduced to Pt(II) form after 21 days in the darkness. The incorporation of the π-acceptor ligand led to a redshift of the absorption peak of the LMCT band to 289 nm, resulting in a high absorption coefficient of the complex in the UVA region. The photoreduction of complex **7** and following binding to GMP was observed only 1 min after UVA irradiation. Further mechanism studies of the photoreaction revealed that each azide ligand gave one electron to the Pt(IV) center to reduce the Pt(IV) to the active Pt(II) species [46]. The activity of complex **7** against a series of cancer cell lines was tested to measure the cytotoxicity and specificity. Compared with cisplatin, the photocytotoxicity of complex **7** increased 80- and 15-fold in A2780 and A2780cisR cells, respectively. Moreover, complex **7** did not show any cross-resistance with cisplatin or oxaliplatin, suggesting its unique mode of action. The incubation of **7** with CT-DNA in solution led to 50% Pt bound to DNA after 1 h irradiation, and the value reached a plateau of 87% after 5 h continuous irradiation. In an in vitro DNA damage repair synthesis assay in HeLa cells, the plasmid platinated by **7** under irradiation induced lower levels of DNA repair synthesis compared with those of cisplatin or transplatin. This might account for the remarkable photocytotoxiciy of complex **7**. In addition, the accumulation of p53 protein was not detected in cells treated with **7** under irradiation, which was different from that of cisplatin. The in vivo antitumor activity of **7** was further investigated in nude mice bearing OE19 xenografted tumors [46]. The results confirmed that **7** had visible light-induced augmented antitumor activity, and the mice can tolerate **7** at a high dose (10 times of the maximum tolerated dose of cisplatin). Therefore, complex **7** was a highly potent photoactivatable prodrug with low in vitro and in vivo toxicity, higher photocytotoxicity, and distinct mechanism of action.

The above-mentioned complexes were activated by UV light, which meant they had poor tissue penetration depth, limiting the clinical application. To further extend the spectrum of activation wavelength, complex **8** (Figure 2) was prepared by replacing the two amine groups with pyridines [47]. Complex **8** had low-intensity absorption in the blue region, which was attributed to mixed LMCT and IL (interligand) transitions. The photoactivation of complex **8** led to a dissociation of azide and formation of Pt(II) containing two pyridines, which subsequently bound to 5′-GMP to form monofunctional crosslinks. Complex **8** exhibited remarkable photocytotoxicity against a panel of human cancer cell lines under irradiation with visible light (λ = 420 nm). In HaCaT cells, the IC_50_ value of complex **8** under a low dose of blue light irradiation was 9.5 μM, which was an order of magnitude more potent than that of cisplatin. Mechanism studies revealed that the photoproduced products of complex **8** efficiently bound to nuclear DNA and formed conformationally varied DNA adducts, which was different from that of cisplatin or transplatin [48]. The adducts stalled RNA polymerase II and inhibited RNA synthesis at a high level compared with cisplatin. Later research found that aizdyl radicals were also generated during the photodecomposition of complex **8** by spin trapping using 5,5-dimethyl-1-pyrroline *N*-oxide (DMPO) [49]. The azidyl radicals also contributed to the unique mode of cell killing. Quenching of the azidyl radicals by l-tryptophan decreased the photocytotocity of complex **8**. These properties made complex **8** a solid visible-light activatable prodrug.

### 2.3. The Derivatives of Diazido Pt(IV) Photoactive Complexes

**Figure 3:**
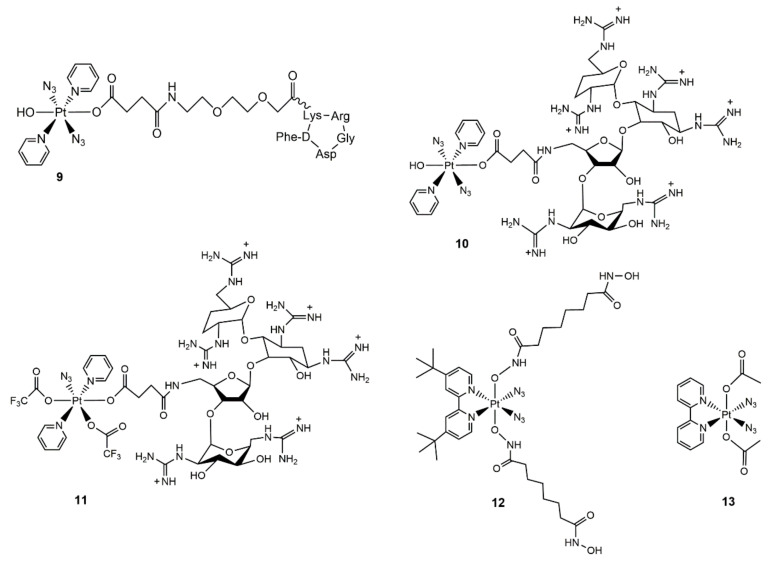
Chemical structures of diazido Pt(IV) derivatives.

The success of the diazido Pt(IV) photoactivatable prodrug stimulated scientists to further optimize them to enhance the cancer cell-targeting properties or cytotoxicity. Complex **8** was the most potent one, so several derivatives were developed based on it. Marchan et al. conjugated a cyclic pentapeptide containing the RGD sequence c(RGDfK) to the axial OH group of complex **8** by using succinic acid as a linker to yield the Pt(IV)–peptide conjugate complex **9** (Figure 3). The c(RGDfK) is capable of binding to the α_v_β_3_ integrin receptor, which is overexpressed in several cancer cell lines. The irradiation of complex **9** in the presence of 5′-GMP led to the formation of Pt(II)–GMP as the major product, which was similar to that of complex **8**. This result suggested that the esterification of the axial OH group did not alter the photochemical properties of complex **8**. Complex **9** showed 4-fold higher cytotoxicity in SK-MEL-28 cells (integrin receptor-positive human melanocarcinoma) compared with in DU145 cells (integrin receptor-negative human prostate cancer cells) under irradiation with blue light (λ = 420 nm). Further studies verified that the accumulation of platinum in SK-MEL-28 cells was higher than that in DU145 cells after treatment with complex **9**. This work highlighted that the cancer-targeting group was able to enhance the selectivity of the photoactivatable diazido Pt(IV) complexes. Lately, the same research group designed complex **10** (Figure 3) by replacing the c(RGDfK) peptide in complex **9** with guanidinoneomycin [50]. Guanidinoneomycin is a known RNA binding ligand and is able to transport bioactive molecules into cells in a proteoglycan receptor-dependent manner. The guanidinoneomycin vector might potentially lead to the platination of RNA over DNA by complex **10** under irradiation, which will result in a novel mechanism of action. The photoactivation of complex **10** in the presence of 5′-GMP mirrored the results of the parent complex **8** with the formation of Pt(II)–GMP adducts. The incorporation of guanidinoneomycin enhanced the cellular accumulation of the Pt(IV) prodrug in the SK-MEL-28 melanoma cells (about 4-fold) compared with the parent complex **8**. However, the photocytotoxicty of complex **10** in SK-MEL-28 cells was similar to that of the parent complex **8**, indicating the different mechanisms of action for complexes **10** and **8**. Notably, the replacement of an azido group and the axial hydroxy group by trifluoroacetate was observed during the step-wise synthesis of complex **10**. The trifluoroacetated analog **11** (Figure 3) also formed Pt–GMP adducts in the presence of 5′-GMP under visible light irradiation. This accidental finding provided a new strategy for the design of novel monoazido-containing Pt(IV) photoactivatable complexes.

The conjugation of a bioactive ligand at the axial position of Pt(IV) prodrugs has been extensively studied as a strategy to potentiate the anticancer activity of platinum drugs, and the drugs were known as “dual-action” Pt(IV) prodrugs [51,52,53,54,55,56]. Recently, Kasparkova et al. prepared complex **12** (Figure 3) by conjugating a histone deacetylase (HADC) inhibitor, suberoyl-bis-hydroxamic acid (Sub), to the axial position of the diazido Pt(IV) complex [57]. The inhibition of HDAC will induce the hyperacetylation of histone proteins, leading to an increased accessibility of chromosomal DNA by platinum(II) drugs and consequently enhanced anticancer activity. Complex **12** showed elevated photocytotoxicity in both cisplatin-sensitive A2780 cells and cisplatin-resistant A2780cisR cells compared with cisplatin or its photoactivatable Pt(IV) derivatives containing inactive axial ligands (complex **13,** Figure 3), confirming the important role of the Sub ligand. Mechanism studies indicated that photoproduced Pt(II) species bound to genomic DNA, and the concomitant released Sub ligand accelerated the DNA damage. The treatment of A2780 cells with complex **12** in the darkness decreased the HDACs activity, and the HADCs activity was further inhibited by complex **12** under irradiation with UVA or blue light. In addition, the presence of a bulky *t*Bu_2_bpy group effectively blocked the RNA polymerase II activity, and photoactivated complex **12** formed more inter-strand cross-links (ICLs) compared with cisplatin. ICLs are considered to be the most lethal lesions among the various Pt–DNA adducts. All of the above-mentioned behaviors of complex **12** contributed to its higher toxicity.

### 2.4. Other Photoreduction Activatable Pt(IV) Complexes

**Figure 4:**
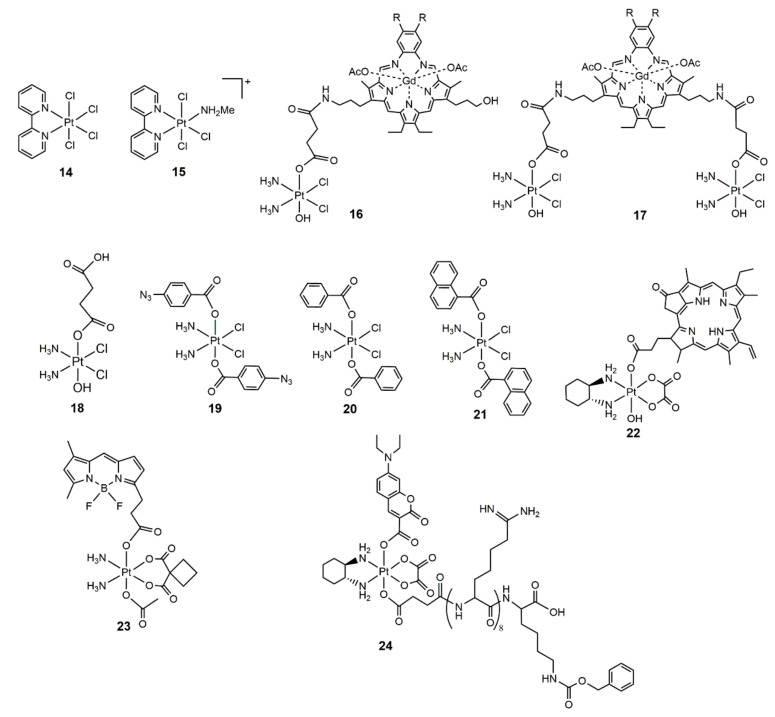
Chemical structures of photoreduction Pt(IV) complexes **14**–**24.**

In the studies of oxidation of nucleobases by Pt(IV) complexes, Pt(IV) chloro complexes **14** and **15** (Figure 4) bearing a 2, 2′-bipridine (2,2′-byp) ligand were designed on the consideration that the use of 2,2′-byp will lead to more positive redox potential [58]. Surprisingly, they found that complexes **14** and **15** underwent photoreduction to Pt(II) species by daylight irradiation, and the major product was [Pt(X)(MeNH_2_)(2,2′-bpy)]^+^(X = Cl or OH). The formed [Pt(Cl)(MeNH_2_)(2,2′-bpy)]^+^ readily bound to the N7 position of guanine in the presence of 5′-GAGCTG [59]. Notably, hypochlorous acid was formed simultaneously in the photoreduction process, which might induce oxidative damage to biomolecules. However, complexes **14** and **15** underwent the hydrolysis of Cl^-^ and isomerization under irradiation and reduction upon heating (50 °C) for 24 h. Hence, the dark stability of this type of complex needed to be further optimized. In order to develop the tumor-targeting ability and to control the platinum reactivity, Sessler et al. prepared Pt(IV)–texaphyrin conjugates **16** and **17** (Figure 4) by conjugating gadolinium texaphyrin, a class of tumor localizing agents, to the precursor Pt(IV) complex **18** (Figure 4) [60]. Complex **18** was reduced to Pt(II) species and bound to 5′-GMP to form [Pt(5′-GMP)_2_(NH_3_)_2_]. Upon irradiation with glass filtered daylight for 2 days, conjugate **16** inherited the photoreduction property of complex **18**. When the mixture of complex **16** and CT-DNA was irradiated, 8.5 ± 1.1 times more Pt–DNA adducts were formed than that in the darkness. Both complexes **16** and **17** showed comparable antiproliferative activity in cisplatin-sensitive A2780 and cisplatin-resistant A2780CP cells. However, the photocytotoxicity of these conjugates toward cancer cells was not tested. Recently, based on the possibility of hemolytic photocleavage of the axial Pt–O bonds within a Pt(IV) prodrug, Ang et al. developed complexes **19**, **20**, and **21** (Figure 4) containing aryl ligands. The ligands were chosen based on the assumption that the departing radical species upon photocleavage were able to be stabilized [61]. A UV (365 nm) irradiation of complex **19** led to the evolution of N_2_, and cisplatin was detected in the precipitate. The irradiation of complex **20** with UV light resulted in the formation of cisplatin and the benzoyl radicals. The latter was quickly quenched by the solvent to yield benzoic acid. A photoreaction of complex **20** with dGMP was further investigated, and complex **20** was unable to react with dGMP in the darkness, while mono-dGMP and bis-dGMP adducts were formed after exposure to UV light for 1 h. The photo behavior of complex **21** was similar to that of complex **20**. This work demonstrated a novel approach to develop photoactivatable Pt(IV) prodrugs. However, more work is needed to advance this strategy to cellular assay.

Tetracarboxylato Pt(IV) complexes were generally stable against cellular reductants. A photo-absorber that can transfer electrons to the Pt(IV) center under irradiation might accelerate the reduction of tetracarboxylato Pt(IV) prodrugs. Based on this hypothesis, our group developed a red light activatable Pt(IV) prodrug, phorbiplatin (**22**) (Figure 4), by conjugating a photosensitizer pyropheophorbide A (PPA) to the axial position of the oxaliplatin-based Pt(IV) scaffold [62]. PPA is capable of catalyzing redox reactions via electron transfer under irradiation and has been used as a photosensitizer in photodynamic therapy. As anticipated, phorbiplatin was stable in the darkness even in the presence of ascorbate, with 89% of the complex kept intact after 24 h incubation. Meanwhile, 81% of phorbiplatin was reduced under irradiation with a lower power density of red light (650 nm, 7 mW/cm^2^) for 10 min to release oxaliplatin and PPA. Mechanism studies and density functional theory (DFT) calculation suggested that the PPA moiety within phorbiplatin was reduced to its π radical anion under irradiation, and the formed π radical anion further transferred an electron to the Pt(IV) center to initiate the reduction process. Phorbiplatin was non-toxic toward A2780 and A2780cisR cancer cells (IC_50_ > 10 μM) in the darkness. When irradiating the treated cells with red light, the IC_50_ values of phorbiplatin in A2780 cells and A2780cisR cells were 0.13 ± 0.01 μM and 0.19 ± 0.01 μM, respectively, which showed 500- and 1000-fold higher cytotoxicity compared with that of oxaliplatin. Further studies verified that both the released oxaliplatin and ROS generated from the PPA moiety contributed to the amazing photocytotoxicity. Owing to the deep tissue penetration of red light, phorbiplatin achieved increased antitumor activity and reduced toxicity in mice bearing 4T1 breast cancer. This work provided a new strategy for designing photoactivatable Pt(IV) prodrugs. Lately, a green light activatable carboplatin-based Pt(IV) prodrug, BODI–Pt(IV) (**23**) (Figure 4), was designed by using the same strategy [63]. In BODI–Pt(IV), carboplatin was used instead of oxaliplatin, due to the fact that carboplatin-based Pt(IV) scaffolds are more inert than the oxaliplatin-based ones. Boron dipyrromethene (bodipy) was used as the photosensitizer for the photoactivation of Pt(IV). As expected, the BODI–Pt(IV) was stable even in the presence of ascorbate but could be quickly reduced to carboplatin under irradiation with green light (13 mW/cm^2^). BODI–Pt(IV) showed elevated cytotoxicity toward cancer cells than carboplatin and was non-toxic to normal cells in the darkness. For instance, in MCF-7 cells, the IC_50_ value of carboplatin was 642.6 ± 51.4 μM, while the value was 15.7 ± 1.0 μM for BODI–Pt(IV) with irradiation, which showed 38.7-fold enhanced cytotoxicity compared with that of carboplatin. Mechanism studies indicated that BODI–Pt(IV) preferentially accumulated in the cytoplasm and nucleus, arrested cell cycle at the G2/M phase, generated ROS, and induced oncosis. This work further confirmed that tethering a photo-absorber to the axial position of an inert Pt(IV) prodrug was an effective approach for the construction of photoactivatable Pt(IV) prodrugs.

Nuclear DNA is believed to be the main target of platinum drugs, thus, the development of photoactivatable prodrugs that targeted the nucleus is of particular interest. Very recently, Zhu et al. designed a nucleolus targeting Pt(IV) complex, coumaplatin (**24**) (Figure 4), which contained an inert oxaliplatin-based Pt(IV) scaffold, a coumarin dye as the fluorophore for photoinduced reduction of Pt(IV), and a cell-penetrating peptide R_8_K [64]. Coumaplatin was very stable in the darkness with 90% of the complex remaining in the cells after 12 h treatment. Upon blue light irradiation, coumaplatin was reduced and released oxaliplatin. Interestingly, the reduction of coumaplatin was associated with the oxidation of water and subsequent evolution of oxygen, which was a novel photoreduction mechanism for Pt(IV) prodrugs. Coumaplatin preferentially accumulated in nucleoli and displayed two orders of magnitude higher photocytotoxiciy than that of oxaliplatin. Intriguingly, coumaplatin induced cell senescence, p53-independent cell death, and immunogenic cell death, which killed cancer cells via a distinct mode of action. This work not only proved a new strategy for the design of visible light activatable Pt(IV) prodrugs but also highlighted that a shift of the subcellular distribution of oxaliplatin to the nucleus will lead to a unique mode of action.

## 3. Photo-Uncaging Pt (II) Complexes

**Figure 5:**
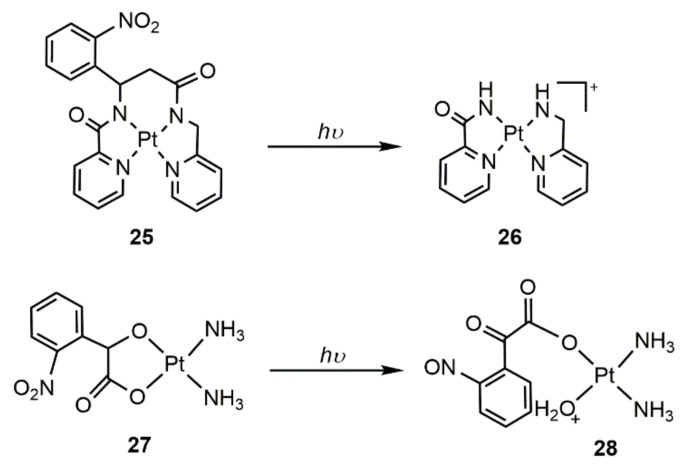
Chemical structures of photo-uncaging complexes **25** and **27** and their formed active species **26** and **28.**

An alternative way for designing photoactivatable complexes is to pre-block the activity of Pt(II) complexes by using photoactivatable groups. Photo-cleavable nitrophenyl groups have been utilized for blocking the biological activity of biomolecules, and UV-light exposure would lead to recovery of the activity. Inspired by this, a novel photocaged Pt(II) complex (**25**) (Figure 5) containing a nitrophenyl group in the ligand backbone was prepared [65]. Under UVA irradiation for only 180 s, the nitrophenyl group was completely cleaved, and a new Pt(II) complex **26** (Figure 5) was formed. Complex **25** was non-toxic to human breast carcinoma MCF-7 cells; however, its cytotoxicity increased by 65% upon UV irradiation. Based on the photoactivity of the nitrophenyl group, Guo and He et al. also built a photoactivatable Pt(II) complex (**27**) (Figure 5) by substituting the labile chloride in cisplatin with 2-hydroxy-2-(2-nitrophenyl)acetic acid [66]. Upon UV irradiation, the nitro group within **27** will be reduced to nitroso, and the hydroxyl group will be oxidized to the carbonyl group simultaneously, resulting in the formation of a new Pt(II) complex **28** (Figure 5)**.** Complex **27** showed enhanced cytotoxicity toward MCF-7 cells under UV irradiation. For example, the inhibition rate in the darkness was 3% at 10 μM treatment with complex **27**, while the value was 18% with UV irradiation. These works developed a new way to build photoactivatable Pt(II) complexes with rapid photoactivation speed. However, the activation required UV light irradiation, which limited the further application due to the short tissue penetration depth of UV light and tissue damage caused by UV light.

## 4. Photodissociation Pt(II) Complexes

**Figure 6:**
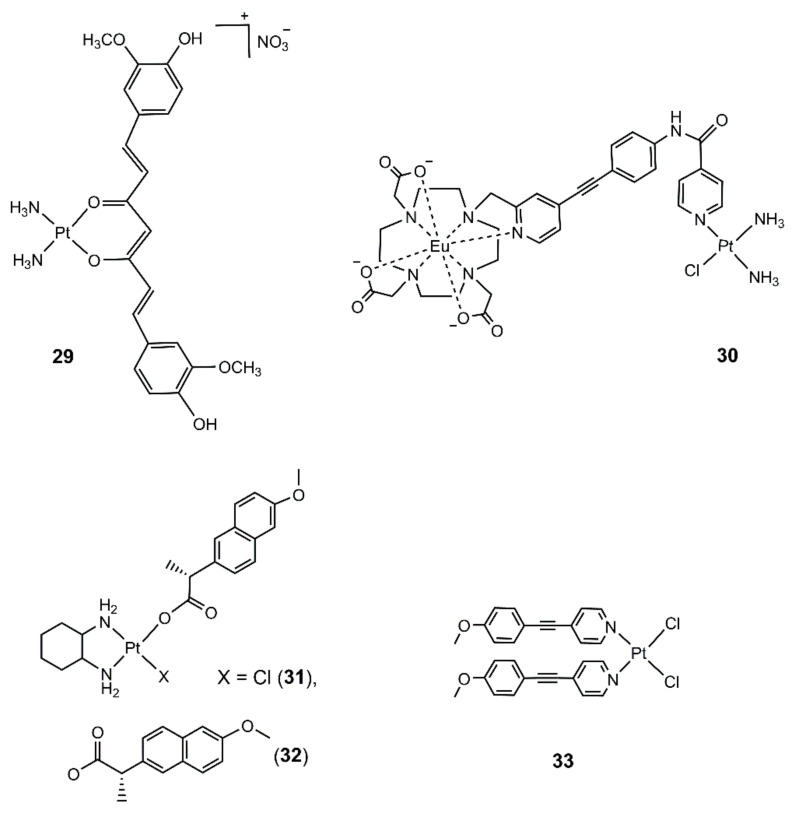
Chemical structures of photodissociation Pt(IV) complexes.

Carboplatin was reported to show an enhanced platination of DNA under UV irradiation. It was suspected that the Pt-O bond was ruptured under irradiation. Thus, the coordination of Pt(II) with an *O*-donor ligand might result in photoactivatable Pt drugs that would release Pt(II) active drugs via breaking of the Pt-O bond under irradiation. Based on this assumption, a platinum (II) complex (**29**) (Figure 6) was prepared by chelating the active form of cisplatin with the enolate form of curcumin [67]. Complex **29** was stable in the darkness, while curcumin was dissociated from **29** under visible light irradiation at 430 nm, and an active form of cisplatin was generated concomitantly. Complex **29** showed a photo-induced formation of Pt-GMP adducts in the presence of 5′-GMP. In contrast, a small portion of complex **29** reacted with 5′-GMP in the darkness after 30 h of incubation. Complex **29** efficiently inhibited the growth of a panel of cancer cells with IC_50_ values of 12–18 μM under irradiation and were non-toxic in the darkness. The remarkable photocytotoxicity was attributed to DNA damage caused by active cisplatin moiety and ROS generated from curcumin. Further fluorescence microscopy showed that complex **29** was located in the cytoplasm and was stable in cells after 2 h. This work demonstrated a novel approach for the design of visible-light activatable Pt(II) complexes by coordinating an active form of cisplatin with a photo-dissociable ligand.

Lately, a photoactivatable lanthanide–cisplatin complex (**30**), **PtEuL** (Figure 6), was constructed by coordinating the active form of cisplatin, [Pt(NH_3_)_2_Cl]^+^, with an Eu coordination complex containing an *N*-donor (**EuL**) [68]. As the energy absorbed by the antenna ligand was transferred to the nearby Pt(II) center, the emission of **EuL** was quenched within **PtEuL**. Upon UVA irradiation, the Pt moiety was dissociated, and the emission of the **EuL** moiety was recovered. Thus, it enabled real-time monitoring of the photoactivation process. **PtEuL** was able to convert the supercoiled form of plasmid DNA to its open-circular form under irradiation, indicating the double-strand breaks caused by the photo-released [Pt(NH_3_)_2_Cl]^+^. **PtEuL** displayed lower cytotoxicity in A549 and HeLa cells compared with cisplatin in the darkness, suggesting the good dark stability of the complex. However, the photocytotoxicity was not reported in their work. Notably, two-photon excitation at 730 nm induced the photodissociation of **PtEuL** within cells after 30 min irradiation as a longer wavelength excitation increased the penetration depth. This work also proved the photodissociation of a photophysical ligand from cisplatin under irradiation.

By utilizing this strategy, Patra and coworkers designed multi-functional photoactive platinum(II) complexes [69]. Non-steroidal anti-inflammatory drugs (NSAIDs) have shown the ability to potentiate platinum drugs against multi-drug resistance and metastatic tumors. In their design, an NSAID, naproxen, was coordinated to the active moiety of oxaliplatin to yield complexes **31** and **32**. The complexes were stable in Tris-HCl buffer for 8 h, as indicated from UV-vis spectral changes. Upon irradiation with UVA light, the naproxen was gradually released, and the emission of naproxen was recovered. The emission intensity was unchanged in the darkness, which further confirmed that the naproxen was bound with the Pt center in the darkness. However, co-incubation of the complexes with 5′-GMP in the darkness resulted in the formation of a Pt-GMP bi-adduct, [Pt(DACH)(5′-GMP)_2_]^+^ (DACH = (1*R*,2*R*)-1,2-diaminocyclohexane), which implied that the complex would cause DNA damage in the darkness. The presence of naproxen ligand could also induce the photocleavage of DNA under irradiation at 365 nm via the formation of singlet oxygen and hydroxyl radical. Thus, it behaved as a dual-action chemotherapeutic with the simultaneous generation of active oxaliplatin and ROS. The complexes showed comparable cytotoxicity toward HeLa and HepG2 cells to cisplatin in the darkness. Upon irradiation, the cytotoxicity was enhanced by 2-fold. These works further demonstrated that the coordination of a photophysical ligand to the active form of cisplatin or oxaliplatin was able to build photoactivatable Pt(II) prodrugs, but the reaction of these complexes with DNA in the darkness was still a concern.

To construct a photoactivatable Pt(II) complex that can be activated with a longer wavelength, two-photon absorption was involved. Complex **33** (Figure 6) was designed by coordinating a long coplanar π-conjugated ligand 4-[2-(4-methoxyphenyl)ethynyl]pyridine (MOPEP) to the Pt(II) center [70]. In MeCN, the complex was stable in the darkness and upon irradiation with light (λ > 500 nm). Under UV irradiation, the MOPEP ligand was dissociated, and the solvent MeCN molecules were coordinated to the Pt(II) as indicated from changes in UV-vis and ^1^H NMR spectra. When using a focused femtosecond laser pulse at a wavelength of 600–740 nm for two-photon absorption activation of the complex, the observed photoactivation process was the same as that activated by UV light. This work demonstrated a novel way to shift the activation wavelength of Pt complexes to red light by using TPA. However, the solubility of this type of complex might be a concern, and the cellular photocytotoxicity was not reported in their work.

## 5. Conclusions and Perspectives

Photoactivatable platinum-based anticancer drugs represent a new type of anticancer drugs, the cytotoxicity of which could be spatially and temporally controlled by irradiation. Hence, photoactivatable platinum-based anticancer drugs could be site-activated in the tumor area to minimize the side effects of platinum drugs, and the distinct mechanism of action of photoactivatable platinum drugs also hold great promise to overcome cisplatin resistance. In this review, photoactivatable Pt(IV) and Pt(II) anticancer complexes that would generate an active form of Pt(II) species through photo-induced reduction, photo-uncaging, and photodissociation were summarized. The rationale for the drug design and the mechanism of the photoactivation process of these photoactivatable platinum anticancer drugs were discussed. The dark cytotoxicity, the photocytotoxicity, and effects on cellular pathways of typical complexes were demonstrated. Compared with classical Pt(II) drugs, photoactivatable platinum anticancer drugs have many advantages such as reduced cytotoxicity in the darkness, enhanced cytotoxicity under irradiation, circumventing drug resistance, and in situ monitoring of the accumulation and activation of the drugs within cells.

Despite the great anticancer effects of the photoactivatable platinum drugs in cellular assay, only several complexes were tested in vivo, and none of them has entered clinical trials. Challenges remain in building a photoactivatable platinum drug with good aqueous solubility, high dark stability, selective accumulation in a tumor site, and rapid activation by irradiation in therapeutic windows (650–950 nm, NIR region). The currently reported ones only had one or two of these characters. The solubility and stability of Pt prodrugs were highly related with the axial ligand, and it had been shown that Pt(IV) complexes bearing carboxyl ligands in the axial position were generally more stable than those with halides or hydroxyl groups; thus, organic acids might be used as the axial ligand to increase the stability. The tumor-targeting properties of photoactivatable complexes could be achieved by conjugating with the tumor targeting moiety such as peptides, aptamers, and antibodies. As the mechanism of the photoactivation process was less understood, the rationale for tuning the photoactivation speed still needed to be explored. The longer wavelength activation at 650–950 nm could be realized by using two-photon absorption activation, upconversion materials, and NIR fluorescent dyes. Currently, several diazido Pt(IV) complexes could be activated by red light through two-photon absorption, but the activation efficacy was very low. Future work may focus on the improvement of the two-photon absorption cross-section of these complexes. Upconversion material can transfer NIR light to UV or blue light for the activation of Pt drugs, but the upconversion efficiency is very low at this stage. Upconversion materials with higher upconversion efficiency might be used for the activation of Pt drugs in the future. Our research indicated that conjugating a red light absorber to an inert Pt(IV) complex was able to build a red light-activatable platinum drug. Thus, the conjugation of NIR fluorescent dyes with inert Pt(IV) complexes might generate NIR light-activatable Pt drugs. In conclusion, the photoactivatable platinum-based anticancer complexes provide new treatment of cancers with low side effects and novel mechanisms, especially for platinum-resistant cancers. It is worth being further developed to expand their application in the clinic.
